# Once a useful engine: a multilevel ethnographic case study on the transgenerational transmission of migratory trauma in Mexican-American families in a sanctuary city

**DOI:** 10.3389/fpsyt.2025.1638643

**Published:** 2025-11-18

**Authors:** Maria Luisa Fernández-Apan, Liz Hamui-Sutton

**Affiliations:** 1 Universidad Nacional Autonoma de Mexico, Programa de Doctorado en Ciencias Medicas y de la Salud, Mexico City, Mexico; 2 Universidad Nacional Autonoma de Mexico, Facultad de Medicina, Mexico City, Mexico

**Keywords:** transgenerational trauma, migratory trauma, Mexican-American families, extended case study, ethnography, high school, neurodivergence

## Abstract

This article explores migratory trauma from an anthropological perspective, using the case study of Dani, a Mexican-American adolescent diagnosed with autism and anxiety. Through an extended case methodology and the use of ethnographic vignettes, it examines the transgenerational transmission of trauma in migratory contexts, along with the school, linguistic, and emotional barriers migrant families face. The interpretive analysis of Dani ‘s drawing, *Once a Useful Engine*, serves as a central narrative device, symbolizing the tension between agency and structure in the lived experience of trauma. The article offers a situated reading of suffering, grounded in the concepts of memory, structural violence, and the micro, meso, and macro-level dynamics shaping Dani ‘s and his family’s trajectory. Findings underscore the need for culturally responsive intervention models and propose public policy recommendations aimed at addressing the complexity of migrant family experiences in a more integrative and just manner.

## 1 Introduction

Contemporary migration phenomena have sparked increasing interest in understanding how migratory experiences impact the mental and emotional health of individuals and communities involved (1, 2). The study of migratory trauma from an anthropological perspective presents an opportunity to understand the complex interactions between sociocultural contexts and individual experiences during migration. Migrant populations, especially those in situations of social subalternity, face unique challenges that may exacerbate social suffering and the impact of trauma across generations (3–5). Unequal power relations, systemic discrimination, and social marginalization contribute to the construction and perpetuation of migratory trauma, both individually and collectively (6–8).

## 2 Research objectives

This study seeks to examine migratory trauma from an anthropological perspective, with a focus on how subaltern relations and social suffering shape the experience, expression, and recovery of trauma within migratory contexts. Additionally, it aims to identify mechanisms of migratory trauma transmission in order to understand how traumatic experiences are carried forward and transformed within migrant communities. The research adopts an emic perspective, centering the voices of those directly involved through narrative inquiry and situated ethnographic observation.

### 2.1 Theoretical framework

This study is grounded in a critical anthropological framework that conceptualizes trauma not solely as a clinical or individual phenomenon, but as a historical, social, and cultural construct. Migratory trauma is understood as a multidimensional experience rooted in structural violence, forced displacement, vulnerabiliity, and systemic barriers to accessing basic rights. Drawing on Kleinman et al. (2) notion of social suffering, the analysis highlights how institutional and societal structures intensify human pain. It also incorporates theoretical contributions on inter- and transgenerational trauma (9), as well as Veena Das’s work on the everyday as a space where suffering is processed and rearticulated, and Spivak’s insights into subaltern agency and silenced voices.

### 2.2 Justification for the multilevel approach

To deepen the understanding of migratory trauma and its transmission, this study employs a multilevel analytical framework that integrates micro, meso, and macro dimensions. At the micro level, it explores familial and individual experiences where trauma is lived and transmitted through intimate relationships, personal narratives, and affective processes. As Das (4) argues, trauma narratives are often fragmented and embedded in specific contexts, where silences and omissions play a central role in the articulation of suffering. These situated narratives provide insight into how individuals negotiate, resist, and re-signify traumatic experiences, developing coping strategies within their relational and social environments.

The meso level focuses on the role of social institutions—such as schools, health centers, and community organizations—that mediate personal experiences and shape conditions of inclusion or exclusion. This dimension examines the resources, protocols, and institutional practices that influence the well-being of migrant families and the care of neurodivergent children (10, 11).

At the macro level, the analysis considers the broader social, political, and economic structures that frame the lives of migrant populations. These include immigration policies, structural discrimination, power dynamics, and legal frameworks that either restrict or facilitate access to fundamental rights (2, 12). This level underscores how systemic forces generate and perpetuate inequalities that shape migrants’ mental health outcomes and educational trajectories.

This multilevel perspective aligns with Bronfenbrenner’s ecological model (10) and draws from critical medical and social anthropology, offering a transdisciplinary framework that goes beyond the juxtaposition of different disciplines. In line with Nicolescu (13) Morin (14), and Darbellay (15), transdisciplinarity opens a space of integration where scientific, clinical, and experiential knowledge converge to generate new epistemological and methodological perspectives. This approach enables a situated, relational, and holistic analysis that acknowledges both the agency of individuals and the structural constraints that condition their lives.

## 3 Literature review

Contemporary research has highlighted the numerous educational and cultural barriers encountered by migrant families, especially those raising neurodivergent children. Researchers have underscored the insufficient preparedness of school systems to effectively respond to the cultural and functional diversity present in these contexts (16, 17), along with the enduring effects of unresolved parental trauma on the mental health of subsequent generations (18). Nevertheless, there is a persisting gap in the literature regarding integrative frameworks that connect neurodiversity, mental health, migration, and anthropological insights to more comprehensively address the layered challenges faced by these families.

### 3.1 Mechanisms of trauma transmission in Dani’s case

Key mechanisms of transgenerational trauma transmission identified include:

• Silences and narrative fractures within the family nucleus, generating affective voids and lack of knowledge about family history, limiting identity construction and trauma understanding.
• Maternal emotional overload stemming from previous violence and abuse, reflected in hypervigilance, guilt, and difficulties establishing secure affective bonds with Dani.
• Linguistic and cultural barriers hindering communication and mediation with school institutions, increasing feelings of isolation and disconnection.
• Relational patterns marked by control and domestic violence affecting family dynamics and children’s emotional safety perceptions.

These complex mechanisms interact and perpetuate the traumatic experience across generations, necessitating integral and contextualized approaches.

In recent years, a growing body of work has expanded the understanding of migratory trauma through anthropological and social-scientific perspectives. Studies highlight the structural determinants of refugee and migrant mental health (19) and frame immigration itself as a social determinant of health (20). Additional contributions from transcultural psychiatry, notably the research of Kirmayer et al. (21), emphasize the interplay between cultural context, resilience, and family narratives. These perspectives inform the present study’s transdisciplinary framework and situate migratory trauma within broader social and institutional processes that reproduce vulnerability across generations.

## 4 Methodology

The design is based on the extended case as a way to understand the articulation between individual experiences (micro level) and social structures (macro level), including educational, health, and legal institutions. This approach situates the analysis within a dynamic, processual, and structural network. The strategy privileged an emic, situated, and collaborative perspective recovering participants’ voices inscribed in a structural understanding framework. Integrating clinical tools with narrative resources allowed access to silence zones, reinterpretation, and symbolic expression of migratory trauma and its generational transmission.

The methodological strategy applied an anthropological approach combining ethnography and the extended case methodology (22) to analyze how migratory trauma is interpreted and transmitted intergenerationally in Mexican families in a sanctuary city.

This proposal maintains an open dialogue with critical medical anthropology and Latin American qualitative research. Ethnography is conceived not only as an observational technique but as a form of relationship, attentive listening, and sustained accompaniment enabling access to subjective experiences and resignifications of involved subjects. This methodology was designed with an open and processual logic, inspired by Loza Taylor (23) and the theoretical-methodological proposal for analyzing suffering narratives developed by Hamui Sutton (24). As Veena Das (4) notes, situated narratives do not simply recount events but allow access to meanings and senses emerging in intersubjective and structural contexts, showing how suffering and social experience are resignified in everyday life.

While the study draws on ethnographic principles, it does not constitute full ethnographic fieldwork. Rather, it employs an ethnographically informed clinical approach, combining therapeutic observation, reflective fieldnotes, and narrative interpretation. This approach privileges attentiveness, relationality, and sustained engagement with participants, reflecting an *ethnographic sensibility* toward lived experience rather than a strictly observational design.

## 5 Case study

Dani^1^ is a 15-year-old adolescent diagnosed with autism and anxiety. He lives with his parents and siblings in a predominantly migrant neighborhood in Cicero, Illinois. Dani initiated individual therapy after reporting suicidal ideation. Nearly three years ago, upon entering treatment, he was hospitalized following a suicide plan that involved throwing himself onto the train tracks that cross through his neighborhood—a moment that marked the onset of his therapeutic process. This crisis reflects the severity of his emotional distress, stemming from anxiety, academic pressure, and the absence of adequate support networks.

Over the course of comprehensive treatment—which included individual therapy, psychiatric care for anxiety management, and family involvement in community support sessions—Dani gradually developed the capacity to express his deep emotional pain in more coherent and symbolic ways. His mother, a survivor of sexual violence in Mexico, has experienced high levels of emotional overload and historically limited access to mental health care.

Dani’s father, who suffered a stroke, has a history of emotional control and communication difficulties within the family, but no record of domestic violence. Dani has expressed feeling emotionally depleted, fearful of failure, and disconnected from both his family and cultural identity. Despite strong academic performance, he experiences recurrent episodes of anxiety, withdrawal, and emotional crises.

Clinical work revealed fragmented personal narratives, a lack of transgenerational storytelling, and a pervasive sense of affective emptiness, exacerbated by school-related stress. Over time, Dani ‘s family environment showed notable improvement. His mother’s participation in community support services enhanced her ability to manage emotional stress and more effectively support her son. Although the father did not engage in individual therapy, he participated in several family and community support sessions, where he expressed sadness about his struggles as a provider, fear of failing his family, and communication barriers—both with Dani, due to language differences, and with his wife, with whom he had frequent conflicts. These moments of emotional recognition and expression within therapeutic spaces contributed to a more open and collaborative family dynamic, facilitating Dani ‘s therapeutic progress.

### 5.1 Ethnographic vignette

In March 2025, Dani ‘s school held its end-of-semester exhibition for the “Art and Expression” class, an annual event where students showcase their work. Dani had invited us to attend and present his artwork. The auditorium entrance was guarded with strict security resembling an airport, and the hallways were filled with proud and nervous teenagers. Very few parents were present; Dani’s parents could not attend due to work commitments, although his mother had brought him and planned to pick him up later. Dani was at one of the last booths selling his black ink drawings. His booth displayed four black ink compositions depicting railroads, locomotives, and intricate urban backgrounds. Each drawing was meticulously shaded; one portrayed a train emerging from darkness, another intertwined mechanical lines that resembled veins or roots. He greeted us with a mix of relief and nervousness. Dani ‘s art teacher remarked she had never seen him present his creative process with such clarity and confidence, an evident change that had grown over time and effort.

### 5.2 Interpretative analysis and link to transgenerational transmission

**Figure 1:**
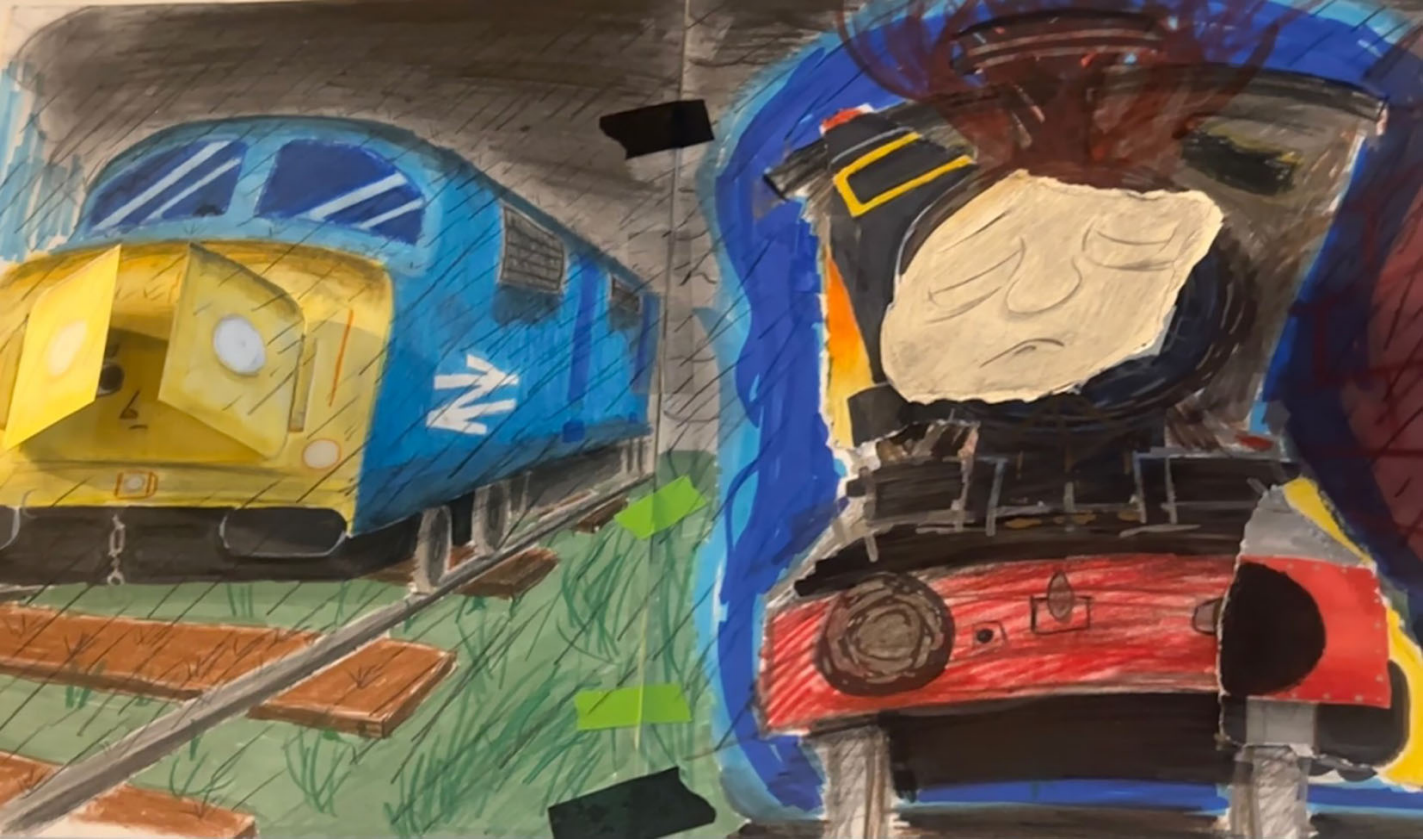
*Once a Useful Engine* Courtesy of Dani, 2025.

Dani titled his drawing *Once a Useful Engine* (**Figure 1**), referring to how he sees himself and his past capacity to cope with school and life demands. This title reflects the tension between his previous sense of usefulness and current fatigue, like a locomotive that has lost strength but must keep moving. The drawing depicts two locomotives on a rainy railroad track. The first represents an early moment in his process, showing a shy, hidden human face in a new, young train. The second is a later version, a close-up with a tired face, more disordered lines, and intervened paper symbolizing the weight and wear Dani intended to convey. According to him, these locomotives represent the train passing in front of his neighborhood in Cicero, a concrete symbol present in his daily life. Beyond its symbolic dimension, the two trains also mirror *Dani’s* own transition from childhood to adulthood. The first, neatly drawn and contained, evokes the child’s need for order and approval; the second, looser and darker, reveals the turbulence of adolescence—an “in-between” state suspended between dependence and autonomy, between past and future. This liminal phase, where identity and belonging are negotiated, reflects both developmental and cultural crossings typical of bicultural youth.

The train serves as a powerful metaphor for both the sense of progress and the emotional weight Dani has endured—a young locomotive tasked with carrying an ever-growing load of responsibilities. This symbol acquires deeper resonance when considering that nearly three years ago, Dani was hospitalized due to suicidal ideation involving a plan to jump onto the Cicero train tracks—a critical moment that marked the beginning of his mental health treatment.

The analysis of Dani’s drawing evokes the genealogy of this image. In many U.S. schools—particularly within migrant communities—the logic of productivity is instilled early: students are expected to advance, produce, comply, improve, and excel. The classroom thus becomes an extension of the industrial assembly line. Since the Industrial Revolution, as Marx (1867/1999) observed, labor has ceased to be a creative endeavor and has become an obligation dictated by performance. This logic is faithfully reproduced in high school, where each student is expected to function as a nascent enterprise. Dani frequently expressed his fear of failure and of not achieving economic success in the future. This anxiety is a concrete expression of transgenerational trauma, with the drawing serving as a symbolic language that articulates the dialectical tension between agency and structure. The worn-out train metaphor reflects Dani’s lived experience: a subject forced to keep moving forward and carry increasing burdens despite emotional and physical exhaustion. This interpretation is supported by the intergenerational patterns observed in Dani’s family. His parents’ narratives reveal inherited expectations of productivity, economic survival, and moral worth that trace back to their own migratory struggles. These ideals, transmitted through affective silences rather than explicit discourse, reappear in Dani’s fear of failure and exhaustion. Several times Dani mentioned feeling ashamed of “being a burden to his family”. Thus, his anxiety does not emerge in isolation but reflects the internalization of generational histories shaped by precarity and the demand to remain “useful” in an unequal system.

While deeply personal, the locomotive image also resonates with a broader social imaginary rooted in the Industrial Revolution, a time when machines became icons of progress and productivity. Yet, as Marx (25) critically analyzed, this progress was inseparable from exploitation and alienation—processes through which labor was mechanized, human subjects were dehumanized, and individuals were reduced to cogs in a productive machine.

Thus, the fatigued and worn locomotive depicted in the drawing symbolizes the unbearable weight of being ‘useful’ or productive within a system that prioritizes performance over holistic well-being. This critique of a utilitarian conception of labor resonates in contemporary analyses, such as those by Byung-Chul Han (26), who argues that violence and self-exploitation in the name of performance have become internalized and self-regulated by individuals. The image becomes a powerful metaphor for the social and structural pressures that affect the mental and emotional health of neurodivergent migrant youth like Dani —who are caught between relentless demands for efficiency and the deep need to sustain a voice and sense of identity.

Dani’s experience also invites reflection on **neurodiversity** as a determinant of migratory trauma. His diagnosis of autism shaped the ways he perceived, processed, and expressed distress. Heightened sensory awareness, literal communication, and difficulties decoding social expectations often intensified the anxiety produced by migratory and educational pressures. At the same time, his artistic production offered an alternative communicative channel, allowing symbolic elaboration of emotions that verbal discourse could not convey. Considering neurodiversity as part of the analytical framework clarifies how neurological difference interacts with migration and structural violence, generating both vulnerability and unique forms of agency.

Dani ‘s artistic expression reveals an ongoing process of working through and giving form to a distress that was previously difficult to articulate. This capacity for expression was made possible through therapeutic engagement, psychiatric support that helped manage his anxiety, and community-based sessions that included his parents. The drawing brings to light the internalized familial and social expectations, deeply entangled with his family’s migratory history. These wounds are not merely individual—they are inherited and shaped by intergenerational dynamics, including maternal emotional overload, paternal control, and chronic communication barriers.

From a reflexive perspective, the therapeutic process also evoked countertransference reactions marked by protectiveness and quiet frustration. At times, Dani’s withdrawn silence produced a sense of helplessness, while his drawings elicited admiration and tenderness. These affective resonances help illuminate how emotions circulate between clinician and adolescent, revealing transgenerational dynamics not only through words but also through embodied sensations.

## 6 Multilevel analysis: micro, meso, and macro

The analysis of Dani and his family’s experience is articulated at three interrelated levels allowing an understanding of migration experience complexity and its multigenerational effects: micro, meso, and macro. This approach enables a situated, deep reading recognizing personal, institutional, and structural dimensions shaping trauma experience and transmission. A multilevel analysis, supported by Bronfenbrenner (10) and Loza Taylor (23) approaches, situates Dani ‘s individual experience within a broader social fabric, facilitating a dialectical understanding of agency and structure in migration experience transmission.

### 6.1 Micro level: family dynamics and personal experiences

At the micro level, Dani ‘s story reveals a profound generational disconnect characterized by narrative and emotional fractures affecting communication and mutual support within the family nucleus. Dani ‘s parents, Mexican migrants whose life trajectories involve violence, abuse, and socioeconomic precariousness (4, 8), face significant difficulties understanding and sustaining Dani ‘s emotional distress and neurodivergence. The mother, a sexual abuse survivor with limited English proficiency, experiences emotional overload marked by hypervigilance and guilt, directly impacting her capacity for stable, secure affective accompaniment. The absence of a shared family narrative and lack of ancestral history knowledge represent identity voids affecting Dani’s sense of self (23). Intergenerational communication gaps and silences linked to historical trauma create shadow zones in family memory, hindering symbolic and emotional elaboration of migration experience (9). These intimate relational conditions are not only the primary channel for migratory experience transmission but also the site where subjects exercise agency, facing and resignifying experiences in social subordination contexts (3, 4).

Dani’s narrative fractures manifest in various family and personal experience aspects. For instance, Dani does not know his grandparents’ names, his parents’ exact birthplace, or have clear references about uncles or cousins. This gap in family history transmission generates disconnection and identity isolation, lacking a shared narrative to situate himself in a lineage or understand his distress’s origin. Additionally, his parents avoid discussing their migratory past and traumatic experiences in Mexico, further deepening the absence of a common narrative framework. These fractures hinder coherent identity construction and limit Dani ‘s trauma integration, perpetuating silences and emotional tensions within the family nucleus. Thus, the micro level offers an essential dimension to understand how transgenerational trauma manifests and reproduces in daily interactions, shaping both risks and possibilities for recovery and affective reconstruction.

### 6.2 Meso level: institutions and social mediation

The meso level has been further developed to examine how schools and community-based organizations act as social mediators in the lives of migrant families. This layer explores how institutional practices, educational protocols, and access to mental health care shape the lived experience of trauma and its intergenerational transmission. By bridging the micro (individual/family) and macro (policy/structural) levels, the meso level underscores the importance of educational institutions as both sites of exclusion and potential spaces of repair. At the meso level, the family confronts a fragmented institutional network insensitive to its complex needs. The local school, a central mediator in Dani ‘s educational and social experience, lacks adequate resources, including competent interpreters and personnel specifically trained in migratory trauma and neurodivergence care (11, 16). Mental health services, primarily Medicaid-funded, offer care that is not always culturally relevant or accessible, limiting intervention effectiveness (27). Furthermore, poor coordination among educational institutions, social services, and health centers generates a fragmented response perpetuating exclusion and the anxiety and vulnerability cycle among migrant families (8). The institutional discourse naturalizing migration experience invisibilizes transgenerational trauma’s impact, which remains hidden in administrative routines and rigid protocols (4). The disconnection between institutional demands and lived realities contributes to migrant neurodivergent students’ emotional and cultural needs going unmet, deepening social and educational exclusion (16, 17). Therefore, the meso level reveals a crucial yet insufficient social mediation space where institutions must reformulate practices to comprehensively respond to these families’ complexity.

### 6.3 Macro level: political, social, and structural context

The macro level encompasses the political and social framework that shapes the lives of migrant families. Illinois, as a sanctuary state, offers certain legal protections; however, the uncertainty and stigma arising from restrictive federal immigration policies create an environment of fear and distrust. Economic precariousness, racial discrimination, and institutional marginalization form a structural context that reproduces inequalities and limits access to fundamental rights such as education and mental health services. The migration background of Dani ‘s parents, which includes dangerous border crossings and experiences of exclusion, is an integral part of this panorama, highlighting the need to address migratory trauma from a comprehensive and political perspective.

The political and economic context in the United States is characterized by persistent anti-immigrant discourses and policies that, along with the global capitalist model, create a context of structural vulnerability for migrant communities. According to Sassen (28), contemporary migration is closely linked to the demands of global capitalism, which requires a flexible and precarious labor force. Migrants, often inserted into informal and poorly regulated sectors, face conditions of labor exploitation, low wages, and lack of social protection (29). This economic phenomenon is exacerbated by social marginalization and racialization promoted by restrictive immigration policies and exclusionary public discourses, deepening systemic exclusion (8, 30). The difficulties of Dani ´s parents to attend and access their child´s school activities, events or academic perform can be framed under these exclusion.

This situation fits within what Galtung (31) terms structural violence, a form of social suffering that impacts negatively the mental health and wellbeing of migrant families, limiting their possibilities for integration and emotional recovery. Thus, migratory trauma cannot be understood without recognizing the political and economic conditions that generate and perpetuate it. As Sen (32) points out, development and wellbeing must be conceived as processes involving freedom and the capacity of individuals to act and rebuild their lives in just and equitable contexts. The invisibilization and criminalization of migration hinder this reconstruction, creating additional barriers to trauma recovery and family agency.

Therefore, the macro level reveals how social and political structures produce and reproduce inequalities that directly impact the mental health and educational trajectory of youth like Dani and their families, highlighting the urgency of inclusive policies that recognize and mitigate these forms of structural violence.

## 7 Public policy recommendations

The current political climate in the United States—characterized by the resurgence of conservative immigration policies and the rise of anti-immigrant rhetoric under the leadership of Donald Trump—has heightened feelings of threat and uncertainty within migrant communities. In sanctuary states such as Illinois, legislative measures have been implemented to safeguard the rights of migrant families. However, poor coordination across different levels of government has led to legal and operational gaps, undermining the protection of fundamental rights such as access to education and mental health services. The criminalization of migration, the persistent threat of deportation, and institutional exclusion foster dynamics of silence, social withdrawal, and distrust that profoundly impact the everyday lives and psychoemotional well-being of migrant children. Recognizing migration experience as a structural determinant of health is an urgent step toward building more just, inclusive, and culturally responsive systems of care.

Drawing from the analysis of Dani and his family, several public policy recommendations are proposed across multiple levels of intervention. At the school level, it is crucial to enhance both emotional and educational support by allocating dedicated resources to accompany neurodivergent students within migrant communities. Priority should be given to the promotion of transdisciplinary teams with training in trauma, migration, and mental health, as well as to the development of flexible protocols that take into account each student’s cultural background and family context. Simultaneously, fostering cultural competence among health and education professionals emerges as key. This involves including ongoing training in cultural diversity, migratory trauma, and transgenerational mental health within curricula and professional development, as well as incorporating bilingual and bicultural personnel in schools and community health centers, ensuring more sensitive and effective care.

At the macro level, migration policies must be grounded in human rights and public health frameworks, ensuring access to education and mental health services for all migrant families, regardless of their legal status. It is also imperative to implement legal frameworks that acknowledge migratory trauma as a social determinant of health, thereby establishing the foundation for more inclusive and equitable public policies. Finally, promoting intersectoral strategies that foster collaboration among educational institutions, mental health services, community organizations, and governmental actors is essential. Developing comprehensive care models that integrate psychosocial support, legal guidance on migration, and school-based accompaniment is crucial for addressing the complex and interconnected needs of migrant families.

These proposals seek to transform the structural conditions that perpetuate the suffering of migrant children, fostering safe, empathetic, and culturally responsive environments that enable families to rebuild themselves through agency and hope. Dani’s case underscores the urgent need to understand child mental health through a lens that integrates transgenerational trauma, neurodivergence, and the systemic barriers migrant families encounter. The anthropological approach adopted here not only renders suffering visible but also highlights the agency and strategies of family reorganization that emerge within highly vulnerable contexts. These elements must be integrated into public policy design, particularly in settings where the well-being of immigrant families remains persistently threatened by political and social instability.

Finally, in direct relation to the study’s central theme of transgenerational trauma, these policy recommendations aim to interrupt the reproduction of inherited distress across generations. Programs that integrate mental-health services with school-based and community interventions can foster early identification of intergenerational patterns of anxiety and silence. Strengthening cross-sector collaboration between educators, clinicians, and community leaders is essential to transforming the institutional environments that sustain these cycles.

## 8 Conclusion

This study has demonstrated how migratory trauma is expressed and transmitted through fragmented narratives, intergenerational silences, and structural tensions that shape migrant families’ life trajectories within contexts marked by inequality. The inclusion of an ethnographic vignette enriched the case’s situated dimension, revealing not only the effects of trauma on the educational experience of a neurodivergent youth, but also the forms of agency and reconstruction that emerge in unexpected spaces, such as a school art fair.

By adopting an anthropological, qualitative, and context-sensitive approach, this research moved beyond traditional clinical interpretations of suffering to foreground processes of meaning-making, expression, and relational accompaniment. Rather than portraying Dani as a bearer of diagnostic labels, he is understood as a subject enmeshed in a historical, affective, and political fabric—where his expressions, such as his artwork, embody not only distress, but also creativity, critique, and the desire to be seen.

The findings emphasize the urgent need to develop public policies and clinical practices that acknowledge the complexity of these trajectories and integrate mental health, education, and social justice. Dani’s case is not an exception; it echoes the experiences of countless children navigating institutional demands, migratory memory, and the struggle to maintain a voice in environments that rarely listen. As such, this study calls for attention not only to systemic shortcomings, but also to the gestures of presence, resistance, and tenderness that arise at the margins—gestures that demand new ways of seeing, accompanying, and building community.

## Data Availability

The datasets presented in this article are not readily available because Interviews were transcripted but are not available due to ethical reasons. Requests to access the datasets should be directed to luisafernandex@gmail.com.

## Footnotes

1 The name *Dani* is a pseudonym used to protect confidentiality.
